# Integration of Ethylene and Light Signaling Affects Hypocotyl Growth in *Arabidopsis*

**DOI:** 10.3389/fpls.2017.00057

**Published:** 2017-01-24

**Authors:** Yanwen Yu, Rongfeng Huang

**Affiliations:** ^1^Biotechnology Research Institute, Chinese Academy of Agricultural SciencesBeijing, China; ^2^National Key Facility of Crop Gene Resources and Genetic ImprovementBeijing, China

**Keywords:** hypocotyl elongation, ethylene signaling, light signaling, seedling emergence, transcriptional activation, protein stability

## Abstract

As an ideal model for studying ethylene effects on cell elongation, *Arabidopsis* hypocotyl growth is widely used due to the unique characteristic that ethylene stimulates hypocotyl elongation in the light but inhibits it in the dark. Although the contrasting effect of ethylene on hypocotyl growth has long been known, the molecular basis of this effect has only gradually been identified in recent years. In the light, ethylene promotes the expression of *PHYTOCHROME INTERACTING FACTOR 3* (PIF3) and the degradation of ELONGATED HYPOCOTYL 5 (HY5) protein, thus stimulating hypocotyl growth. In the dark, *ETHYLENE RESPONSE FACTOR 1* (ERF1) and *WAVE-DAMPENED 5* (WDL5) induced by ethylene are responsible for its inhibitory effect on hypocotyl elongation. Moreover, CONSTITUTIVE PHOTOMORPHOGENIC 1 (COP1) and PHYTOCHROME B (phyB) mediate the light-suppressed ethylene response in different ways. Here, we review several pivotal advances associated with ethylene-regulated hypocotyl elongation, focusing on the integration of ethylene and light signaling during seedling emergence from the soil.

## Introduction

As cell division rarely occurs in the *Arabidopsis* hypocotyl, this system is considered an ideal model for studying cell elongation (Vandenbussche et al., 2005; Boron and Vissenberg, 2014). The hypocotyl is highly responsive to both internal and external cues, such as plant hormones, light, temperature, and gravity (Vandenbussche et al., 2005; Van de Poel et al., 2015). Among these growth regulators, ethylene is special because of its contradictory effect on hypocotyl elongation (Ecker, 1995; Smalle et al., 1997). In the light, the application of ethylene or its precursor 1-aminocyclopropane-1-carboxylic acid (ACC) stimulates hypocotyl elongation, whereas in the dark, ethylene suppresses hypocotyl growth (Zhong et al., 2012; Yu et al., 2013). Additionally, this phenotype suggests a close relationship between ethylene and light signaling in hypocotyl growth.

Ethylene signaling starts with endoplasmic reticulum (ER)-located ethylene receptors (Hua and Meyerowitz, 1998). In the absence of ethylene, ER membrane-located ethylene receptors such as ETHYLENE RESPONSE 1 (ETR1) interacts with and activates the Ser/Thr kinase CONSTITUTIVE RESPONSE 1 (CTR1), which further phosphorylates another ER membrane-located protein ETHYLENE INSENSITIVE 2 (EIN2) (Kieber et al., 1993; Alonso et al., 1999; Ju et al., 2012). The downstream transcription factors EIN3 and EIN3-LIKE 1 (EIL1) are degraded through the F-box proteins EIN3-BINDING F BOX PROTEIN 1 (EBF1) and EBF2, leading to interruption of the ethylene-induced transcription cascade (Chao et al., 1997; Guo and Ecker, 2003; Potuschak et al., 2003). In the presence of ethylene, the interaction of ETR1 with ethylene molecules deactivates CTR1 and leads to the cleavage of unphosphorylated EIN2 (Ju et al., 2012; Qiao et al., 2012). As a result, a portion of the cleavage product, EIN2C, shuttles into the nucleus to activate the EIN3/EIL1-dependent transcription cascade, while the remaining EIN2C is retained in the cytoplasm and inhibits the translation of EBF1 and EBF2 by binding to their mRNAs (Ju et al., 2012; Qiao et al., 2012; Li et al., 2015; Merchante et al., 2015).

Light is not only an energy source but also one of the most important environmental cues for plant growth and development (Chen et al., 2004). Light signaling is perceived by various photoreceptors and leads to the modulation of downstream transcription factors such as PHYTOCHROME INTERACTING FACTORs (PIFs) and HYPOCOTYL 5 (HY5) (Lau and Deng, 2010). For example, light promotes the translocation of the red photoreceptor phyB into the nucleus to directly interact with PIFs, resulting in PIF phosphorylation and degradation (Lau and Deng, 2010; Leivar and Monte, 2014; Ni et al., 2014). In addition, light reduces the level of nuclear-localized COP1 protein and promotes the stabilization of its target protein HY5 (Osterlund et al., 2000). A recent study proposed that the binding of phyB to SPA inhibits the activity of COP1 (Sheerin et al., 2015). Finally, the protein levels of PIFs and HY5 co-determine the transcription level of genes related to seedling photomorphogenesis in the light (Lau and Deng, 2010).

Hypocotyl length changes dramatically in the early plant growth stage, especially between seed germination and seedling establishment. Recently, some studies investigating the underlying mechanisms of seedling emergence have been published and drawn great attention to this stage (Zhong et al., 2014; Shi et al., 2016a,b). Before emerging from the soil, *Arabidopsis* seedlings undergo skotomorphogenesis with closed and pale cotyledons, an apical hook and a fast-growing hypocotyl in the absence of light. Once they emerge from the soil, seedlings adopt photomorphogenesis with open and green cotyledons, especially a shortened hypocotyl (Zhong et al., 2014). Hypocotyl elongation during seedling emergence involves numerous plant hormone responses to external circumstances, which are coordinated via various pathways. Here, we present an overview of ethylene function during hypocotyl elongation, focusing on the interaction between ethylene and light signaling, especially during seedling emergence.

## Ethylene has Differing Effects on Hypocotyl Growth

**FIGURE 1:**
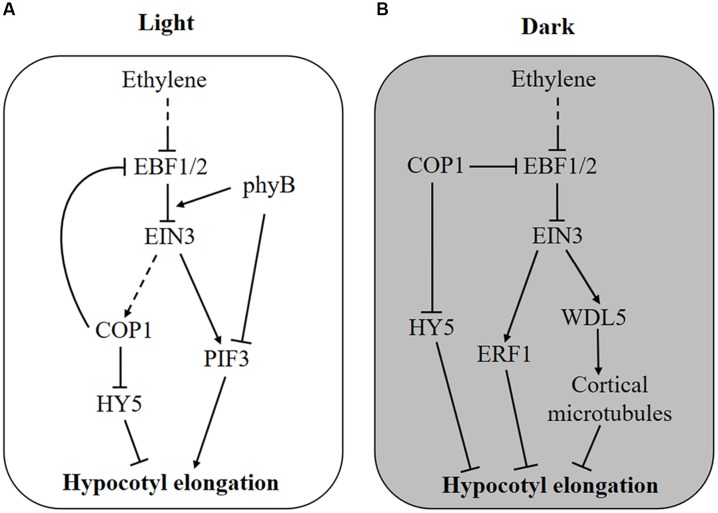
**Molecular mechanism of ethylene-regulated hypocotyl growth. (A)** In the light, ethylene stimulates hypocotyl elongation via transcriptional activation of *PIF3* and degradation of HY5 protein by promoting the enrichment of nuclear COP1 protein. Feedback is probably generated as COP1 stabilizes EIN3 by degrading the F-box proteins EBF1 and EBF2. Additionally, light-activated phyB attenuates ethylene responses by promoting the interaction of EBF1/2 and EIN3. **(B)** In the dark, COP1 is enriched in the nucleus and promotes the degradation of HY5 and hypocotyl elongation, whereas ethylene counteracts this process by activating the expression of *ERF1* and *WDL5*. When seedlings grow toward the soil surface, COP1 and ethylene production are suppressed by increased light and reduced mechanical pressure, respectively, removing the inhibitory effect of EIN3 on hypocotyl elongation.

Ethylene can promote or suppress *Arabidopsis* hypocotyl elongation depending on light conditions (Ecker, 1995; Smalle et al., 1997). In the dark or in low light intensities (<10 μmol/m^2^/s), ethylene acts as a repressor of hypocotyl elongation, whereas in high light intensities or in days with more than 8 h of light, ethylene becomes an activator (Zhong et al., 2012). Furthermore, it was shown that ethylene can promote hypocotyl growth only in red light, not in far red or blue light (Shi et al., 2016b). The function of ethylene in hypocotyl growth is reflected in ethylene mutants as well. For example, ethylene overproduction mutants (*eto1/2/3*) and an ethylene constitutive-response mutant (*ctr1*) show shortened hypocotyls in the dark but elongated ones in the light, and the hypocotyls of ethylene-insensitive mutants (*etr1*, *ein2*, and *ein3 eil1*) exhibit a certain level of shortening in the light (Smalle et al., 1997; Alonso et al., 1999; Zhong et al., 2012; Yu et al., 2013; Shi et al., 2016b). In recent years, several light-signaling elements, including PhyB, PIF3, COP1, and HY5, have been identified to mediate ethylene-regulated hypocotyl elongation (Zhong et al., 2012, 2014; Yu et al., 2013; Shi et al., 2016a,b; **Figure 1**). These findings enable us to better understand how ethylene fine tunes hypocotyl growth under such complicated external environments.

The adverse effect of ethylene on hypocotyl elongation is mediated by the transcription factors PIF3 and ETHYLENE RESPONSE FACTOR 1 (ERF1), which promote hypocotyl elongation in the light and inhibit it in the dark, respectively (Zhong et al., 2012). Although both PIF3 and ERF1 are transcriptionally activated by ethylene through EIN3, their total protein level determines the differing effects of ethylene on hypocotyl growth. In the light, PIF3 protein is rapidly degraded by LIGHT-RESPONSE BRIC-A-BRACK/TRAMTRACK/BROAD (LRB) E3 ligases, whereas ERF1 protein is maintained at a high level (Zhong et al., 2012). Hence, ERF1 induced by ethylene does not function due to an excessive protein level, but PIF3 is sensitively affected by ethylene. Conversely, in the dark, PIF3 protein is saturated, and ethylene-induced ERF1 inhibits hypocotyl elongation (Zhong et al., 2012). Therefore, both ethylene-induced transcriptional control and light-regulated protein accumulation are required in this process. This result might explain why ethylene-promoted hypocotyl elongation requires a certain quantity of light. Recently, another EIN3 target gene, WAVE-DAMPENED 5 (WDL5), was reported to mediate ethylene-inhibited etiolated hypocotyl elongation (Sun et al., 2015; Ma et al., 2016). By binding to cortical microtubules, WDL5 regulates microtubule bundling and microtubule reorientation, finally affecting etiolated hypocotyl elongation (**Figure 1B**). However, the *wdl5* mutant shows a hypocotyl length similar to wild type, regardless of ACC treatment in the light, suggesting that WDL5 does not function in the ethylene-promoted hypocotyl elongation (Sun et al., 2015).

In addition, regulation at the protein level by ethylene has also been found for hypocotyl growth. It was reported that HY5 plays important roles in ethylene-promoted hypocotyl elongation in the light (Yu et al., 2013, **Figure 1A**). HY5 is a native regulator of hypocotyl growth and is controlled at the protein level by the E3 ligase COP1 (Osterlund et al., 2000). In the dark, COP1 is enriched in the nucleus and interacts with HY5 to promote its degradation, leading to an elongated hypocotyl, whereas in the light, COP1 is translocated out of nucleus so that HY5 protein accumulates to inhibit hypocotyl growth (von Arnim and Deng, 1994; Osterlund et al., 2000). Further experiments proved that light-triggered COP1 movement into the cytoplasm is reversed by ethylene, thus, ethylene stimulates hypocotyl elongation by promoting the nuclear localization of COP1 and HY5 degradation (Yu et al., 2013). Genetic experiments have demonstrated that ethylene-regulated COP1 localization and HY5 stabilization is dependent on EIN3 (Yu et al., 2013).

Interestingly, ethylene signaling is also suppressed by light on the protein level (Shi et al., 2016a,b, **Figure 1**). As an E3 ligase, COP1 directly interacts with EBF1/2 and promotes its degradation by the 26S proteasome, leading to the accumulation of EIN3 protein (Shi et al., 2016a). Thus, light can reduce EIN3 stabilization and ethylene signaling through the inactivation of COP1. Interestingly, once ethylene signaling is activated, nucleus-enriched COP1 is also likely to enhance ethylene signaling (**Figure 1A**). More immediately, photoactivated phyB can bind to both EIN3 and EBF1/2, resulting in enhanced interaction between them and EIN3 degradation (Shi et al., 2016b, **Figure 1A**). It is believed that EIN3 plays an important role in the balance between ethylene and light signaling in hypocotyl growth.

## Interactions Between Ethylene and Light Signaling Affect Hypocotyl Growth During Seedling Emergence

Seed plants often start life under the soil. Before reaching the light, germinated seedlings initiate signaling related to darkness and mechanical disturbance (Zhong et al., 2014). The absence of light leads to PIF3 accumulation and nucleus-enriched COP1 but HY5 degradation, both of which lead to an elongated hypocotyl so that the seedling can emerge from the soil quickly (Lau and Deng, 2010; Zhong et al., 2012, 2014). In addition, increased ethylene concentrations induced by the depth and texture of soil inhibit hypocotyl elongation (Zhong et al., 2014). In this situation, ethylene inhibits hypocotyl growth by activating EIN3-mediated ERF1 and WDL5 expression (Zhong et al., 2012; Sun et al., 2015; Ma et al., 2016). The suppression of hypocotyl growth by ethylene leads to a longer period of etiolated growth; thus, PIF3, another target of EIN3, is activated to coordinately regulate chlorophyll synthesis (Zhong et al., 2014). Moreover, the *ein3 eil1* double mutant exhibits a lower survival rate under deep and firm soil cover, further indicating that ethylene-regulated hypocotyl growth and etiolated growth are necessary for seedling emergence from soil (Zhong et al., 2014).

As seedlings grow toward the soil surface, ethylene production is reduced with decreased mechanical stress; meanwhile, the gradual increase in light penetrating through the soil will suppress COP1 activity, thus promoting EBF1/2-mediated EIN3 degradation and relieving the inhibitory effect of ethylene on hypocotyl growth (Shi et al., 2016a). Therefore, COP1 plays dual roles in hypocotyl growth during seedling emergence: Under soil cover, COP1 functions to promote hypocotyl elongation by degrading HY5 protein and enhances ethylene signaling through EBF1/2-mediated EIN3 stabilization (Osterlund et al., 2000; Shi et al., 2016a). As the seedling approaches the soil surface, increasing light relieves COP1-stabilized EIN3 protein and gradually counteracts ethylene signaling (Shi et al., 2016a). Although both ethylene and COP1 affect the stability of EIN3 through EBF1/2, they act independently, because ethylene still promotes the accumulation of EIN3 in *cop1* mutant plants (Shi et al., 2016a).

However, once the seedling breaks through the soil surface, it requires rapid changes, including hook opening and cotyledon expansion, which are counteracted by ethylene signaling (Shi et al., 2016b). At this time, photoactivated phyB functions as molecular glue to promote the interaction between EBF1/2 and EIN3, leading to the degradation of EIN3 and immediate cessation of ethylene signaling (Shi et al., 2016b). The gradual COP1-mediated and fast phyB-dependent regulation of EIN3 protein level guarantee successful seedling emergence from the soil with an appropriate hypocotyl length and accomplish de-etiolation in time.

It has been reported that ethylene production induced by flooding in rice promotes stem elongation above the water level to avoid submergence stress (Hattori et al., 2009). In addition, the positive effect of ethylene on hypocotyl elongation in the light suggests that ethylene is probably still required for hypocotyl growth after seedling emergence from the soil. In the light, ethylene promotes the translocation of COP1 into the nucleus and transcriptionally activates PIF3 expression, attenuating light signaling and thus stimulating hypocotyl elongation (Zhong et al., 2012; Yu et al., 2013). Therefore, the opposing effects of ethylene and light signaling occur throughout hypocotyl growth during seedling emergence.

## Conclusion and Future Perspectives

Ethylene-regulated hypocotyl elongation is a good model for studying how cues from endogenous hormones and environmental factors are integrated in the control of plant growth. This mini review aimed to concisely summarize the crosstalk between ethylene and light signaling in the regulation of hypocotyl growth, focusing on detailing the function of ethylene in hypocotyl growth during seedling emergence. Some factors that were not mentioned here, such as temperature and biological rhythm, are also very important for the regulation of hypocotyl growth.

The antagonistic effect of ethylene on hypocotyl elongation occurs due to the transcription factors ERF1, PIF3, HY5, and WDL5 (**Figure 1**). In the light, ethylene promotes hypocotyl growth at both the transcriptional and the protein level: the transcriptional activation of *PIF3* and the degradation of HY5 protein (Zhong et al., 2012; Yu et al., 2013). In the dark, ethylene suppresses hypocotyl elongation by transcriptionally activating ERF1 and WDL5 via EIN3 (Zhong et al., 2012; Sun et al., 2015). In addition, light functions via COP1 and phyB to promote the degradation of EIN3, which plays important roles during seedling emergence (Shi et al., 2016a,b). Under soil cover, hypocotyl growth is promoted by darkness but inhibited by mechanical pressure-induced ethylene. As seedlings approach and break through the soil surface, the EIN3 level is reduced by increased light via COP1 and phyB, leading to the shutdown of ethylene signaling, which guarantees that seedlings transition from growing in a dark soil cover situation to the bright light soil surface (Shi et al., 2016a,b). After the seedling emerges from the soil, light-suppressed hypocotyl growth is stimulated by ethylene. This process is important for plants to resist submergence stress, because flooding-induced ethylene promotes the elongation of rice internodes to escape from submergence damage (Hattori et al., 2009). However, it is still unknown whether and how ethylene participates in normal hypocotyl growth.

In addition to ethylene, other plant hormones such as auxin, gibberellin (GA), and brassinolide (BR) participate in the regulation of hypocotyl growth. Furthermore, inhibitors of auxin transport (1-N-Naphthylphthalamic acid, NPA), auxin biosynthesis (yucasin), auxin perception (α-(phenylethyl-2-one)-indole-3-acetic acid, PEO-IAA), GA biosynthesis (paclobutrazol, PBZ) and BR biosynthesis (brassinazole, BRZ) or mutants related to these processes suppress ethylene-promoted hypocotyl elongation, suggesting that ethylene regulates hypocotyl growth partly through auxin, GA, and BR (Vandenbussche et al., 2007; Liang et al., 2012; Das et al., 2016). However, the crosstalk between ethylene and plant hormones is different in the light and the dark. For example, in the light, ethylene-promoted hypocotyl elongation can be inhibited by treatment with NPA or in auxin-insensitive mutants, whereas in the dark, ethylene still suppresses hypocotyl growth in these mutants or with NPA treatment (Liang et al., 2012; Das et al., 2016). Therefore, a more complicated regulatory network of plant hormones exists for the regulation of hypocotyl growth.

**FIGURE 2:**
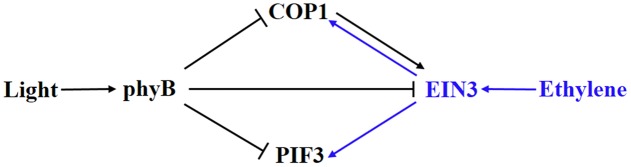
**Involvement of elements in the crosstalk between ethylene and light signaling in hypocotyl growth**. COP1 and PIF3 are suppressed by light via phyB but activated by ethylene via EBF1/2-EIN3. In turn, ethylene-promoted EIN3 is inhibited by light via COP1 and phyB. Blue and dark indicates ethylene and light signaling, respectively.

Ethylene and light converge on EIN3, COP1, and PIF3 in the regulation of hypocotyl growth (**Figure 2**). Ethylene-enhanced EIN3 is inhibited by light through COP1 and phyB. In turn, light-suppressed COP1 and PIF3 can be activated by ethylene (Zhong et al., 2012; Yu et al., 2013; Shi et al., 2016a,b). Therefore, a competitive relationship between ethylene and light signaling is formed by the crosstalk between these elements. An organ-specific, genome-wide investigation of transcriptomic changes in ethylene-promoted and shade-promoted hypocotyl growth demonstrated that a conserved set of transcriptionally regulated genes, especially hormone-related genes, is utilized by plants to modulate hypocotyl growth in response to ethylene and shade (Das et al., 2016). Thus, there might be more elements integrating ethylene and light signaling in hypocotyl growth. Recently, our work indicated that ethylene-promoted COP1 localization in the nucleus affects seed germination under salt stress (Yu et al., 2016), indicating that the interaction between light and ethylene shown in hypocotyl growth probably plays additional roles in other pathways. In any case, the interaction between ethylene and light signaling in hypocotyl growth during seedling emergence is a good example of how plants integrate external signaling and internal hormones.

## Author Contributions

RH proposed the topic. RH and YY collected the literature, critically assessed the information, and wrote the manuscript together.

## Conflict of Interest Statement

The authors declare that the research was conducted in the absence of any commercial or financial relationships that could be construed as a potential conflict of interest.

## References

[B1] Alonso J. M. Hirayama T. Roman G. Nourizadeh S. Ecker J. R. (1999). EIN2, a bifunctional transducer of ethylene and stress responses in *Arabidopsis*. *Science* 284 2148–2152. 10.1126/science.284.5423.2148 10381874

[B2] Boron A. K. Vissenberg K. (2014). The *Arabidopsis thaliana* hypocotyl, a model to identify and study control mechanisms of cellular expansion. *Plant Cell Rep.* 33 697–706. 10.1007/s00299-014-1591-x 24633990

[B3] Chao Q. Rothenberg M. Solano R. Roman G. Terzaghi W. Ecker J. R. (1997). Activation of the ethylene gas response pathway in *Arabidopsis* by the nuclear protein ETHYLENE-INSENSITIVE3 and related proteins. *Cell* 89 1133–1144. 10.1016/S0092-8674(00)80300-1 9215635

[B4] Chen M. Chory J. Fankhauser C. (2004). Light signal transduction in higher plants. *Annu. Rev. Genet.* 38 87–117. 10.1146/annurev.genet.38.072902.092259 15568973

[B5] Das D. St Onge K. R. Voesenek L. A. Pierik R. Sasidharan R. (2016). Ethylene-and shade-induced hypocotyl elongation share transcriptome patterns and functional regulators. *Plant Physiol.* 172 718–733. 10.1104/pp.16.00725 27329224 PMC5047086

[B6] Ecker J. R. (1995). The ethylene signal transduction pathway in plants. *Science* 268 667–675. 10.1126/science.7732375 7732375

[B7] Guo H. Ecker J. R. (2003). Plant responses to ethylene gas are mediated by SCF EBF1/EBF2-dependent proteolysis of EIN3 transcription factor. *Cell* 115 667–677. 10.1016/S0092-8674(03)00969-3 14675532

[B8] Hattori Y. Nagai K. Furukawa S. Song X. J. Kawano R. Sakakibara H. (2009). The ethylene response factors SNORKEL1 and SNORKEL2 allow rice to adapt to deep water. *Nature* 460 1026–1030. 10.1038/nature08258 19693083

[B9] Hua J. Meyerowitz E. M. (1998). Ethylene responses are negatively regulated by a receptor gene family in *Arabidopsis thaliana*. *Cell* 94 261–271. 10.1016/S0092-8674(00)81425-7 9695954

[B10] Ju C. Yoon G. M. Shemansky J. M. Lin D. Y. Ying Z. I. Chang J. (2012). CTR1 phosphorylates the central regulator EIN2 to control ethylene hormone signaling from the ER membrane to the nucleus in *Arabidopsis*. *Proc. Natl. Acad. Sci. U.S.A.* 109 19486–19491. 10.1073/pnas.1214848109 23132950 PMC3511113

[B11] Kieber J. J. Rothenberg M. Roman G. Feldmann K. A. Ecker J. R. (1993). CTR1, a negative regulator of the ethylene response pathway in *Arabidopsis*, encodes a member of the raf family of protein kinases. *Cell* 72 427–441. 10.1016/0092-8674(93)90119-B 8431946

[B12] Lau O. S. Deng X. W. (2010). Plant hormone signaling lightens up: integrators of light and hormones. *Curr. Opin. Plant Biol.* 13 571–577. 10.1016/j.pbi.2010.07.001 20739215

[B13] Leivar P. Monte E. (2014). PIFs: systems integrators in plant development. *Plant Cell* 26 56–78. 10.1105/tpc.113.120857 24481072 PMC3963594

[B14] Li W. Ma M. Feng Y. Li H. Wang Y. Ma Y. (2015). EIN2-directed translational regulation of ethylene signaling in *Arabidopsis*. *Cell* 163 670–683. 10.1016/j.cell.2015.09.037 26496607

[B15] Liang X. Wang H. Mao L. Hu Y. Dong T. Zhang Y. (2012). Involvement of COP1 in ethylene- and light-regulated hypocotyl elongation. *Planta* 236 1791–1802. 10.1007/s00425-012-1730-y 22890836

[B16] Ma Q. Sun J. Mao T. (2016). Microtubule bundling plays a role in ethylene-mediated cortical microtubule reorientation in etiolated hypocotyls. *J. Cell Sci.* 129 2043–2051. 10.1242/jcs.184408 27044753

[B17] Merchante C. Brumos J. Yun J. Hu Q. Spencer K. R. Enríquez P. (2015). Gene-specific translation regulation mediated by the hormone-signaling molecule EIN2. *Cell* 163 684–697. 10.1016/j.cell.2015.09.036 26496608

[B18] Ni W. Xu S. L. Tepperman J. M. Stanley D. J. Maltby D. A. Gross J. D. (2014). A mutually assured destruction mechanism attenuates light signaling in *Arabidopsis*. *Science* 344 1160–1164. 10.1126/science.1250778 24904166 PMC4414656

[B19] Osterlund M. T. Hardtke C. S. Wei N. Deng X. W. (2000). Targeted destabilization of HY5 during light-regulated development of *Arabidopsis*. *Nature* 405 462–466. 10.1038/35013076 10839542

[B20] Potuschak T. Lechner E. Parmentier Y. Yanagisawa S. Grava S. Koncz C. (2003). EIN3-dependent regulation of plant ethylene hormone signaling by two *Arabidopsis* F box proteins: EBF1 and EBF2. *Cell* 115 679–689. 10.1016/S0092-8674(03)00968-1 14675533

[B21] Qiao H. Shen Z. Huang S. S. C. Schmitz R. J. Urich M. A. Briggs S. P. (2012). Processing and subcellular trafficking of ER-tethered EIN2 control response to ethylene gas. *Science* 338 390–393. 10.1126/science.1225974 22936567 PMC3523706

[B22] Sheerin D. J. Menon C. zur Oven-Krockhaus S. Enderle B. Zhu L. Johnen P. (2015). Light-activated phytochrome A and B interact with members of the SPA family to promote photomorphogenesis in *Arabidopsis* by reorganizing the COP1/SPA complex. *Plant Cell* 27 189–201. 10.1105/tpc.114.134775 25627066 PMC4330587

[B23] Shi H. Liu R. Xue C. Shen X. Wei N. Deng X. W. (2016a). Seedlings transduce the depth and mechanical pressure of covering soil using COP1 and ethylene to regulate EBF1/EBF2 for soil emergence. *Curr. Biol.* 26 139–149. 10.1016/j.cub.2015.11.053 26748855 PMC5108888

[B24] Shi H. Shen X. Liu R. Xue C. Wei N. Deng X. W. (2016b). The red light receptor phytochrome B directly enhances substrate-E3 ligase interactions to attenuate ethylene responses. *Dev. Cell* 39 597–610. 10.1016/j.devcel.2016.10.020 27889482 PMC5140706

[B25] Smalle J. Haegman M. Kurepa J. Van Montagu M. Straeten D. V. (1997). Ethylene can stimulate *Arabidopsis* hypocotyl elongation in the light. *Proc. Natl. Acad. Sci. U.S.A.* 94 2756–2761. 10.1073/pnas.94.6.2756 11038610 PMC20163

[B26] Sun J. Ma Q. Mao T. (2015). Ethylene regulates *Arabidopsis* microtubule- associated protein WDL5 in etiolated hypocotyl elongation. *Plant physiol.* 169 325–337. 10.1104/pp.15.00609 26134166 PMC4577400

[B27] Van de Poel B. Smet D. Van Der Straeten D. (2015). Ethylene and hormonal cross talk in vegetative growth and development. *Plant Physiol.* 169 61–72. 10.1104/pp.15.00724 26232489 PMC4577414

[B28] Vandenbussche F. Vancompernolle B. Rieu I. Ahmad M. Phillips A. Moritz T. (2007). Ethylene-induced *Arabidopsis* hypocotyl elongation is dependent on but not mediated by gibberellins. *J. Exp. Bot.* 58 4269–4281. 10.1093/jxb/erm288 18182430

[B29] Vandenbussche F. Verbelen J. P. Van Der Straeten D. (2005). Of light and length: regulation of hypocotyl growth in *Arabidopsis*. *Bioessays* 27 275–284. 10.1002/bies.20199 15714558

[B30] von Arnim A. G. Deng X. W. (1994). Light inactivation of *Arabidopsis* photomorphogenic repressor COP1 involves a cell-specific regulation of its nucleocytoplasmic partitioning. *Cell* 79 1035–1045. 10.1016/0092-8674(94)90034-5 8001131

[B31] Yu Y. Wang J. Shi H. Gu J. Dong J. Deng X. W. (2016). Salt stress and ethylene antagonistically regulate nucleocytoplasmic partitioning of COP1 to control seed germination. *Plant Physiol.* 170 2340–2350. 10.1104/pp.15.01724 26850275 PMC4825130

[B32] Yu Y. Wang J. Zhang Z. Quan R. Zhang H. Deng X. W. (2013). Ethylene promotes hypocotyl growth and HY5 degradation by enhancing the movement of COP1 to the nucleus in the light. *PLoS Genet.* 9:e1004025 10.1371/journal.pgen.1004025 PMC3861121 24348273

[B33] Zhong S. Shi H. Xue C. Wang L. Xi Y. Li J. (2012). A molecular framework of light-controlled phytohormone action in *Arabidopsis*. *Curr. Biol.* 22 1530–1535. 10.1016/j.cub.2012.06.039 22818915 PMC4437768

[B34] Zhong S. Shi H. Xue C. Wei N. Guo H. Deng X. W. (2014). Ethylene-orchestrated circuitry coordinates a seedling’s response to soil cover and etiolated growth. *Proc. Natl. Acad. Sci. U.S.A.* 111 3913–3920. 10.1073/pnas.1402491111 24599595 PMC3964075

